# Comparative Neurodevelopment Effects of Bisphenol A and Bisphenol F on Rat Fetal Neural Stem Cell Models

**DOI:** 10.3390/cells10040793

**Published:** 2021-04-02

**Authors:** Santokh Gill, V. M. Ruvin Kumara

**Affiliations:** Regulatory Toxicology Research Division, Health Products and Food Branch, Tunney’s Pasture, Health Canada, 251 Sir Frederick Banting Driveway, Ottawa, ON K1A 0K9, Canada; ruvin@vidanamadura.net

**Keywords:** brain development, bisphenol, neurotoxicity, stem cell differentiation, regulatory toxicology, oligodendrocytes, neurons, astrocytes, BPA, BPF

## Abstract

Bisphenol A (BPA) is considered as one of the most extensively synthesized and used chemicals for industrial and consumer products. Previous investigations have established that exposure to BPA has been linked to developmental, reproductive, cardiovascular, immune, and metabolic effects. Several jurisdictions have imposed restrictions and/or have banned the use of BPA in packaging material and other consumer goods. Hence, manufacturers have replaced BPA with its analogues that have a similar chemical structure. Some of these analogues have shown similar endocrine effects as BPA, while others have not been assessed. In this investigation, we compared the neurodevelopmental effects of BPA and its major replacement Bisphenol F (BPF) on rat fetal neural stem cells (rNSCs). rNSCs were exposed to cell-specific differentiation media with non-cytotoxic doses of BPA or BPF at the range of 0.05 µM to 100 µM concentrations and measured the degree of cell proliferation, differentiation, and morphometric parameters. Both of these compounds increased cell proliferation and impacted the differentiation rates of oligodendrocytes and neurons, in a concentration-dependent manner. Further, there were concentration-dependent decreases in the maturation of oligodendrocytes and neurons, with a concomitant increase in immature oligodendrocytes and neurons. In contrast, neither BPA nor BPF had any overall effect on cellular proliferation or the cytotoxicity of astrocytes. However, there was a concentration-dependent increase in astrocyte differentiation and morphological changes. Morphometric analysis for the astrocytes, oligodendrocytes, and neurons showed a reduction in the arborization. These data show that fetal rNSCs exposed to either BPA or BPF lead to comparable changes in the cellular differentiation, proliferation, and arborization processes.

## 1. Introduction

Bisphenol A (BPA) is among the 800 chemicals known or suspected to have endocrine-disrupting properties [1,2,3]. It has been used by industry for the synthesis of various polymers, including epoxy resins, polycarbonate, and certain plastics [4,5,6,7]. Human exposure to BPA occurs through multiple sources including food packaging, dust, dental materials, healthcare equipment, thermal paper, and toys and related articles for infants and children [8,9,10].

Clinical, epidemiologic, and experimental data show that exposure to BPA may be linked to adverse health concerns, including neurodevelopment disorders, obesity, cardiovascular disease, reproductive disorders, behavioral effects, diabetes, chronic respiratory failure, kidney disease, and carcinogenesis [11,12,13,14,15,16]. Studies have shown that BPA can cross the placenta and may lead to effects on fetal development after early life exposure [17]. Based on these potential adverse health effects, several jurisdictions (including Health Canada, ESFA, FAO/WHO, 2011; FDA, 2013) have regulated the production, the usage, and the importation of BPA for selected products including food contact material and articles, baby bottles, sippy cups, and infant formula packaging [18,19,20]. Hence, manufacturers have introduced the usage of BPA analogues such as BPAF, BPAP, BPB, BPE, BPF, and BPP into the market [21]. BPB (2,2-bis(4-hydroxyphenylbutane) and BPF are being used for the manufacturing of polycarbonates and epoxy resins [22,23,24]. Although the data available are limited on the occurrence of these bisphenol analogues in foods and human body fluids and tissues, various analogues have been detected in the environment, wildlife, foods or food packaging materials, consumer products, personal care products, and indoor house dust [20,24,25,26,27,28,29,30,31]. Worldwide biomonitoring studies have detected some of these analogues (BPAF, BPB, BPF, BPS) in the serum, breast milk, umbilical cord blood, and urine [22,32,33,34,35,36,37,38]. Although BPA was the most dominant bisphenol in different matrices, some analogues were found in equal or in greater amounts than BPA in some environmental and food samples, which most likely reflects a shift from BPA to other substitutes in some applications [39]. The role of BPA exposure on cellular differentiation and development processes is well documented; however, the effects of some of these analogues, on the differentiation and/or development processes are unknown [40,41,42,43,44]. In vitro studies have shown that some of the BPA analogues have similar or even greater endocrine disruptive activity than BPA [21,45]. Based on these findings, it is important to resolve whether exposure to these BPA analogues, especially during the embryonic period, contributes to comparable neurodevelopmental effects. 

BPF is currently one of the analogues that is increasingly used as a replacement for BPA in the synthesis of polymers required for the production of microwave dishes, baby bottles, artificial organs, dialyzers, coatings, etc. Additionally, it’s a raw material in the production of many products such as developers for heat-sensitive paper, fire retardants, intermediates for colorants, pharmaceuticals, pesticides, etc. [39]. Preliminary evidence indicates that BPF may have a longer duration for elimination and similar estrogenic activity within the body compared to BPA. In addition, BPF has been detected in human bodily fluids and tissues [40,41,42,43,44]. Compared to BPA, there is limited data for the development effects of BPF. Although there are other BPF derivatives such as tetramethyl bisphenol F, tetramethyl bisphenol F diglycidyl ether, and tetramethyl bisphenol F epoxy resin, only BPF was widely detected in human tissues and body fluids such as in serum, breast milk, umbilical cord blood, and urine [35,36,37,38]. Therefore, it is imperative to investigate the potential effects of BPF on neural stem cell differentiation processes and how it may contribute to developmental neurotoxicity. In this study, we compared the effects of non-cytotoxic doses of BPA and BPF on the rNSCs cellular proliferation and differentiation processes including arborization using a monolayer-based system, as previously described [46]. Sholl analysis [47] has previously been used to quantify the arborization for neurons [48,49,50,51,52,53,54,55,56], astrocytes [5,57,58,59,60,61], and oligodendrocytes [48,62,63,64,65]. Briefly, it creates a series of circles with increasing radii centered on the soma and measures the number of time dendrites or cellular process intersects in each circle and plotted as a function of radial distance from the cell soma [47,48,51]. It quantifies branching complexity dendritic or cellular process arbor [55,56,57,64,65] and hence microcircuit structure [51,56,66,67]. We used Fiji-ImageJ [68] software for the Sholl analysis [47,69].

## 2. Materials and Methods

### 2.1. Reagents

Preparation of reagents, handling, and discarding of toxicological and bio-hazardous materials were carried out as recommended by Health Canada’s laboratory safety regulations. Both BPA and BPF were obtained from Sigma-Aldrich, (St. Louis, MO, USA) and a 1 mM stock solution of each was prepared in sterile water and frozen at −20 °C until use. BPA or BPF working dilutions were prepared by diluting stock solution with the relevant differentiation medium. T3 (Thyroxine; Sigma-Aldrich, St. Louis, MO, USA) was dissolved in 1N of NaOH and an aliquot was mixed with sterile culture medium to prepare 20 µg/mL stock solution and frozen at −20 °C until use. Fetal rNSC (Gibco, Life Technologies Corporation, Frederick, MD, USA) and Lab-Tek chamber slide systems (Thermo Fisher Scientific, Waltham, MA, USA) were used throughout as in our previous study for culturing and immunofluorescence staining [46].

The primary antibodies that were used included Anti-Nestin (Abcam, Cambridge, MA, USA), Anti-Glial Fibrillary-Acidic-Protein (GFAP, Abcam), Anti-A2B5 (Abcam), Anti-Galactocerebroside clone-mGalc (Galc, Millipore, MilliporeSigma, Burlington, MA, USA), Microtubule-Associated-Protein2 (MAP2) clone-M13 monoclonal (Invitrogen, Life Technologies Corporation, Carlsbad, CA, USA), MAP2 clone-AP18 (monoclonal, Invitrogen), and Doublecortin (monoclonal, Invitrogen). These primary antibodies were used to detect protein-markers specific to neural stem cells, astrocytes, oligodendrocytes, and neurons, respectively [46].

Several different secondary antibodies were used to attach fluorophore to the primary antibody-protein-marker complex. These included: (a) Goat-Anti-Mouse-IgG-H&L Alexa Fluor-488 (Abcam) was used to detect the Anti-Nestin, Anti-A2B5, MAP2 clone M13 antibodies; (b) The Goat-anti-Mouse-IgG-(H+L)-Alexa Fluor Plus-647 (Invitrogen) was used to detect Doublecortin monoclonal antibody; (c) Goat-Anti-Rabbit-IgG-H&L Alexa Fluor-488 (Abcam) was used to detect Anti-GFAP antibody; and (d) Mouse-IgG (H+L)-cross-adsorbed Alexa Fluor-594 (Invitrogen) was used to detect Anti-Galactocerebroside-clone-mGalc antibody, and MAP2 clone-AP18 monoclonal antibody. 5% Goat serum was used to prepare working solution of all the primary and secondary antibodies [46].

### 2.2. Cell Culture

#### Propagation of rNSCs

For the propagation of rNSC, the culture medium (complete StemPro NSC SFM) was similar to the one used in our earlier publication [46]. It is composed of recombinant human Fibroblast Growth Factor basic (FGFb, 20 ng/mL, Gibco, Life Technologies Corporation, Carlsbad, CA, USA ), recombinant human Epidermal Growth Factor (EGF, 20 ng/mL, Gibco, Life Technologies Corporation, Carlsbad, CA, USA), Glutamax (2 mM 100×), and StemPro Neural Supplement (2% in 1× KnockOut D-MEM/F-12 medium, Gibco, Life Technologies Corporation, Carlsbad, CA, USA). T75 culture flask was coated with 15 mL of Poly-L-ornithine (20 μg/mL, Sigma, Burlington, MA, USA) and incubated overnight at room temperature (RT), rinsed twice with D-PBS (in the absence of Ca^2+^ and Mg^2+^ and used for the rNSC propagations. Multipotent fetal rNSC (Gibco) were subjected to 3-types of the directed-differentiation process to obtained astrocytes, neurons, and oligodendrocytes. The rNSCs were cultured (2 × 10^6^ cells) in the stem cell growth medium in the incubator at 37 °C, 5% CO_2_, and 90% humidity. The following day, the medium was replaced with a pre-warmed stem cell growth medium. The growth medium was changed with a fresh growth medium every 2–3 days, and the cells were passaged when confluency reached 75–90%. Passage number 3 rNSCs (P3) were used for the differentiation experiments [46].

### 2.3. rNSC Monolayer Model for Directed-Differentiation to Generate Astrocytes, Neurons, and Oligodendrocytes

#### 2.3.1. Coating Chamber Slides for 3-Types of Directed-Differentiation Processes

For directed-differentiation of rNSCs into neurons and oligodendrocytes, Poly L-ornithine and Laminin (Gibco, Life Technologies Corporation, Frederick, MD, USA were used successively for double coating the chamber slides. Initially, the surface coating was done with Poly l-ornithine (40 μg/mL, Sigma) and allowed to incubate overnight at room temperature in the laminar flow and then washed twice with sterile water and air-dried. Subsequently, chamber slides were coated second time with Laminin (10 μg/mL, Gibco), and allowed to incubate for 2 h at 37 °C and then rinsed with D-PBS (in the absence of Ca^2+^ and Mg^2+^). For the astrocytes directed-differentiation process, the chamber slides were coated with Geltrex (1×, Gibco, Life Technologies Corporation, Carlsbad, CA, USA) and incubated for 90 min at 37 °C [46].

#### 2.3.2. Differentiating Media

Astrocyte directed-differentiation medium was similar to the one described in Gill and Kumara [46].

Oligodendrocyte specific modified directed-differentiation medium composed of DMEM/F12 (1×, Gibco) supplemented with N-2 supplement (1%, 100×, Gibco) and B27- (2%, 50×, Gibco) of T3 (49 ng/mL, Sigma) and BSA (1%, Thermo Scientific). The introduction of B27 into the medium enhanced the directed-differentiation process of rNSCs into oligodendrocytes [46,70,71].

Neuron directed-differentiation medium contains B27- (2%, 50×, Gibco) Glutamax (2 mM, 100×) in Neurobasal medium (1×, Gibco) [46].

#### 2.3.3. rNSC Culturing for Differentiation

The rNSCs with passage number 3 were cultured approximately 7.80 × 10^4^ cells per chamber in stem cell growth medium and incubated at 37 °C, 5% CO_2_, and 90% humidity. Once the cells were attached to the surface, the medium in the control chambers was changed with fresh stem cell growth medium, whereas for the directed-differentiation of rNSCs into astrocytes, the medium in the slide chambers coated with Geltrex was replaced with the astrocyte-specific directed-differentiation medium. For the directed-differentiation of rNSCs into oligodendrocytes, the medium in the slide chambers coated with poly-l-ornithine and Laminin was replaced with the oligodendrocyte-specific directed-differentiation medium. Similarly, for the directed-differentiation of rNSCs into neurons, the medium was replaced with the neuron-specific directed-differentiation medium. All chamber slides were placed in the incubator adjusted for 37 °C, 5% CO_2_, and 90% humidity. The media were changed with the freshly prepared relevant medium every 2 days and the directed-differentiation process was continued for 7 days. After 7 days, the differentiation media was removed and cells were washed once with PBS. Subsequently, the cells were fixed with 4% PFA for 15 min at RT and then washed three times with PBS in the presence of Ca^2+^ and Mg^2+^. The chamber slides were loaded with 0.5 mL of PBS (with Ca^2+^ and Mg^2+^), sealed with Parafilm and stored at 4 °C for the immunostaining process [46].

### 2.4. Cell Proliferation or Cytotoxicity Assays

The relative cell count (RCC) was used as an index of cell proliferation or cytotoxicity. RCC was expressed as a “Total number of cells % control” and calculated using the following equation. “Total number of cells % control” = (No of nuclei of viable cells in treated group/no of nuclei of viable cells in the control group) × 100, which has been used in the previous publications [46,72,73].

### 2.5. Effects of BPA or BPF on the Differentiation Rate of rNSCs into Astrocytes, Oligodendrocytes, and Neurons

The experiments performed in Section 2.3.3 and Section 2.4 were repeated with a similar dose range of freshly prepared BPA or BPF introduced to each directed-differentiation media in the three differentiation processes. Each directed-differentiation media or growth media were replaced with a freshly prepared relevant medium containing BPA or BPF every two days. The concentrations of both BPA and BPF used were 0.05 μM, 0.25 μM, 10 μM, 50 μM, and 100 μM, in relevant differentiating media directed into astrocytes, neurons, and oligodendrocytes or rNSC in the growth medium. Relevant control groups were treated only with the vehicle that BPA or BPF dissolved. After 7 days, the medium was removed, cells were washed with PBS, and fixed with 4% PFA for the immunostaining and enumeration. DAPI (4′,6-Diamidino-2-phenylindole dihydrochloride) stained nuclei of fluorescent and phase-contrast overlapping images were used for the enumeration of the total number of cells of each cell category. Images were obtained and the total number of nuclei were counted by ImageJ software. The RCC and the %differentiation [74] of rNSCs into astrocytes, oligodendrocytes, and neurons were measured [46,74].

### 2.6. Immunochemistry and Image Analysis

The fixation of cells, staining, and anti-body dilutions was performed as previously described [46]. The fluorescent and phase-contrast images were captured by EVOS FL Color Imaging System equipped with a Sony ICX285AQ color CCD camera, and image analysis was performed as previously described [46].

### 2.7. Morphometric Analysis of Neurons, Astrocyte and Oligodendrocyte Differentiated from rNSC in the Presence and Absence of BPA or BPF

Images of each cell type with specific protein-markers were segmented and transformed into a binary mask before tracing the cellular processes of each cell. The “Simple Neurite Tracer” of Fiji-ImageJ software (National Institutes of Health, Bethesda, MD, USA) [68] was used to trace randomly selected complete individual astrocyte, oligodendrocyte, or neuron cells. Images with those traced cells were subjected to morphometric analysis using Sholl analysis [47,69].

Traced individual whole cells of neurons, astrocytes, and oligodendrocytes differentiated from rNSC in the presence and absence of 0 μM (control), 50 μM, and 100 μM of BPA or BPF, were subjected to Sholl analysis [69] (Sholl analysis version 3.7.0 of Fiji-ImageJ software 1.52i, National Institutes of Health, Bethesda, MD, USA) [68]. Concentric circles were placed around the traced whole-cell starting from the center of the soma and radiating outward at increasing radial increments of 3 μm. The number of intersections was enumerated as points where cellular processes of each cell type cross a concentric Sholl ring. The number of intersections per circle was plotted against radial distance from the soma of each cell type [46].

### 2.8. Statistical Analysis

Normal distribution was examined by the Shapiro–Wilk and Kolmogorov–Smirnov tests were used to check normal distribution. ANOVA (IBM SPSS 19 program, IBM, New York, NY, USA) was used to determine the overall effect of BPA or BPF on the “% differentiation” of rNSCs into neurons, oligodendrocytes, and astrocytes. Levene’s test was used to control the homogeneity of variances. When the Levene’s test is not significant (the homogeneity of variance ensured parameters) two-way ANOVA was used. When the Levene’s test is significant (the homogeneity of variance not ensured) Welch’s ANOVA was used. For the pairwise comparison, the Bonferroni test was used in the cases that the variances are homogenous, and Games–Howell test was used when the variances are not homogenous. These pairwise comparison tests compare the difference between various parameters of the BPA and BPF treated cells as well as relevant controls. All data are represented as means ± standard error (S.E.), and statistical significance was given in the following order, ^+^ *p* < 0.05, ^++,#^ *p* < 0.01, ^+++, ##^ *p* < 0.001, in each experiment legends.

## 3. Results

### 3.1. Effects of BPA and BPF on rNSC Differentiation Directed into Astrocytes

**Figure 1:**
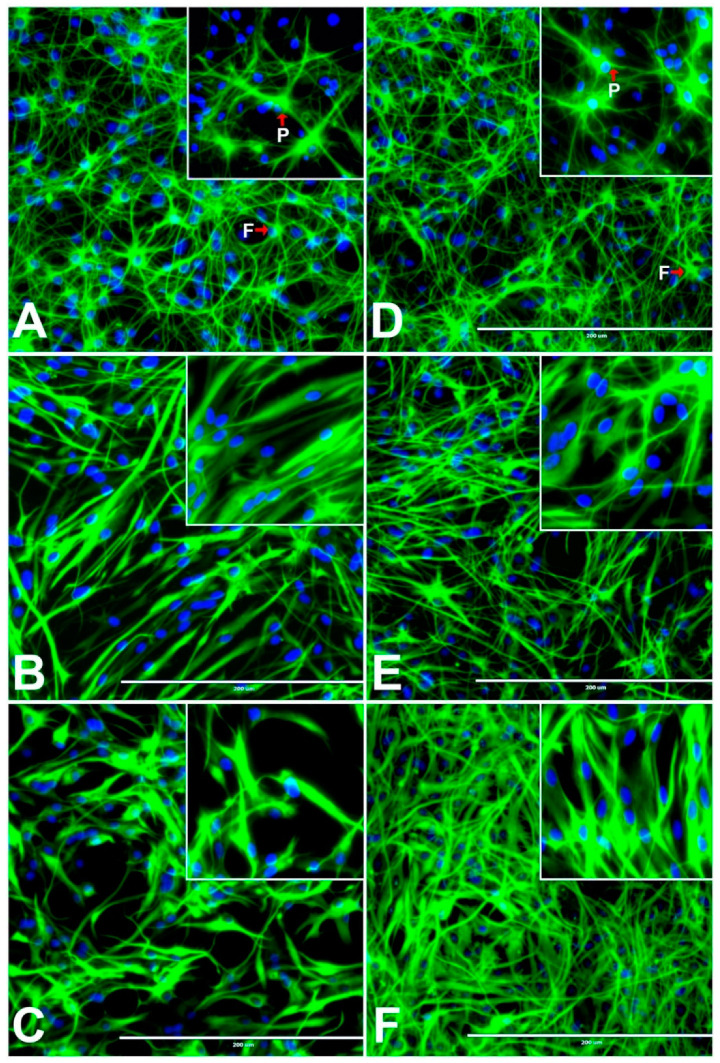
Different concentrations of Bisphenol A (BPA) or Bisphenol F (BPF) affect the morphological differentiation process of rNSCs into astrocytes. The rNSCs grown in astrocyte directed-differentiation media in the presence of or absence of BPA or BPF for a seven day period. Differentiated astrocytes were visualized with astrocyte-specific protein-marker Glial Fibrillary-Acidic-Protein (GFAP) and nuclei fluorescent-marker DAPI. The fluorescent images shown are representative images of astrocyte differentiation (**A**) in the absence of BPA (control), (**B**) in the presence of 50 μM BPA, (**C**) 100 μM BPA; (**D**) in the absence of BPF (control), (**E**) in the presence 50 μM BPF, and (**F**) 100 μM BPF. Arrows indicate protoplasmic (**small P**) and fibrous (**small F**) astrocytes. Scale bar indicates 200 μm at 20× magnification.

**Figure 2:**
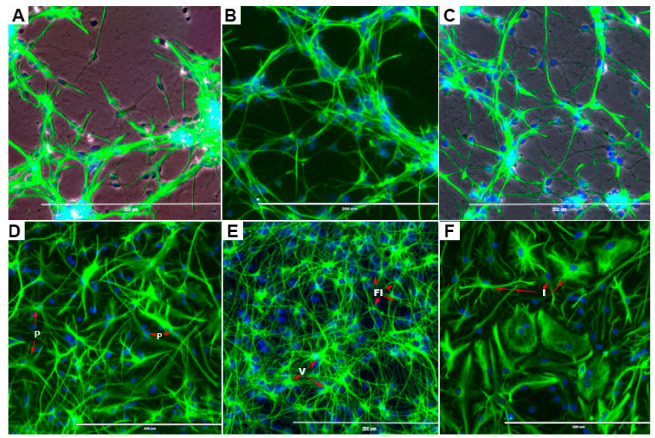
Upper panel images illustrate the effects of BPA or BPF on stem cell properties of rNSCs cultured in NSC growth medium for a seven day period. Subsequently, rNSCs were immunostained with stem cell specific protein-marker Nestin and nuclei stained with DAPI. (**A**) Fluorescent and phase-contrast overlapped image of control rNSC cells. (**B**) Fluorescent image of 50 µM BPF treated rNSCs, and (**C**) fluorescent and phase-contrast overlapped image of 50 µM BPA treated rNSCs. The lower panel illustrates representative images of morphologically different types of astrocytes differentiated from rNSC cultured in astrocyte directed-differentiation medium for seven days in relevant controls. Astrocytes differentiated from rNSCs were immunostained with specific marker GFAP and nuclei stained with DAPI. (**D**) Protoplasmic [P], (**E**) fibrous [FI], and varicose projection [V] and (**F**) interlaminar [I] type astrocytes. Scale bar indicates 200 μm at 20× magnification.

The rNSCs were differentiated into astrocytes using cell directed differentiation media. After seven days of differentiation, four distinct protoplasmic, fibrous, varicose-projection, and interlaminar astrocytes were visualized by immunofluorescent staining with astrocyte cell-specific marker- Glial Fibrillary Acidic Protein (GFAP; Figure 1A,D and Figure 2D–F). Interestingly, in the presence of 50 µM and 100 µM of either BPA or BPF, rNSCs differentiated into fusiform shaped astrocytes with elongated soma and shorter processes (Figure 1B,C,E,F). Additionally, in the presence of 100 µM BPA, rNSCs generated astrocytes that showed complete morphological changes with no process or shorter process, while 100 µM BPF generated astrocytes with abnormal morphology and the four types of astrocytes were no longer visible. The rNSC astrocytes treated with 100 µM of BPA had more drastic morphological changes (Figure 1C) and shorter cellular process from the soma, compared to the 100 µM of BPF treated astrocytes (Figure 1F).

### 3.2. Effects of BPA or BPF on Stem Cell Properties of rNSCs Cultured in rNSC Growth Medium

The rNSCs cultured in NSC growth medium parallel with the 3-types of directed differentiation medium in the presence or absence of 50 µM BPA or BPF for 7 days. rNSCs were immunostained with stem cell specific protein-marker Nestin to investigate the effects of BPA or BPF on stem cell properties. Almost all rNSCs were stained with Nestin in the control group as well as BPA or BPF treated groups indicating that BPA or BPF do not change stem cell marker protein expression and hence stem cell properties (Figure 2A–C).

### 3.3. Effects of BPA or BPF on rNSC Differentiation Directed into Neurons

**Figure 3:**
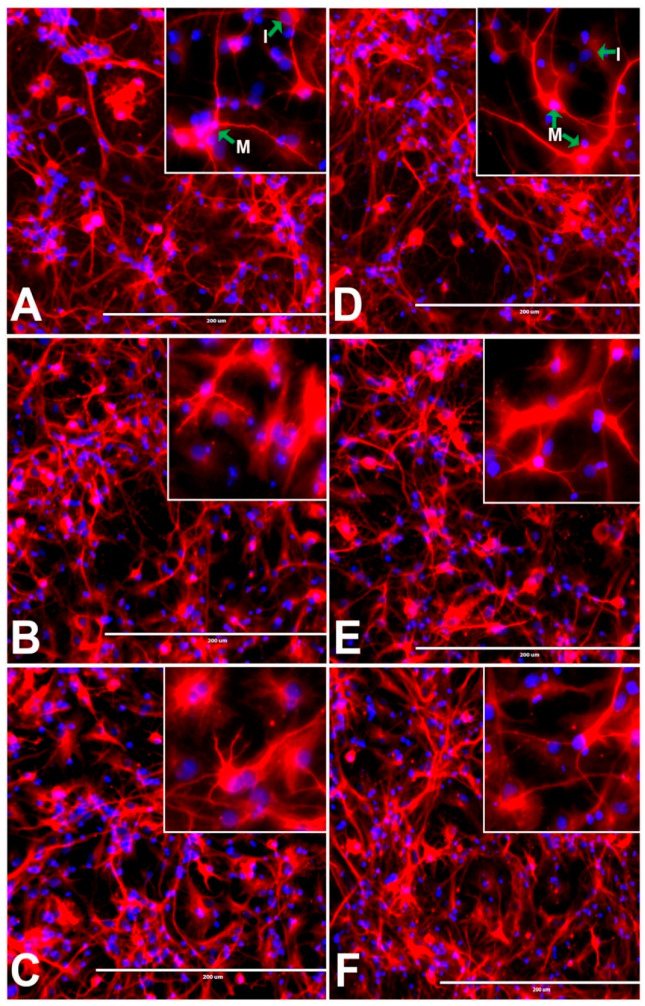
Effects of different doses of BPA or BPF on the morphological differentiation of rNSCs into neurons. rNSCs grown in neuron directed-differentiation media in the absence or presence of BPA or BPF for a seven day period. Differentiated neurons were visualized with specific neuronal protein-marker MAP2 and nuclei marker DAPI. The images shown are representative fluorescent-images of differentiated neurons (**A**) without BPA (control), (**B**) with 50 μM of BPA, (**C**) with 100 μM of BPA; (**D**) without BPF (control), (**E**) with 50 μM of BPF, and (**F**) with 100 μM of BPF. Arrows indicate mature neurons with characteristic morphology (**M**) and immature neurons without characteristic morphology (**I**). Scale bar indicates 200 μm at 20× magnification.

The rNSCs cultured with neuronal differentiation medium for 7 days were differentiated into immature and mature neurons. The mature neurons had different types of axonal morphologies, such as unipolar, bipolar, multipolar, as well as pyramidal-like neurons. These axonal morphologies were detected in phase contrast MAP2 clone-AP18 immunofluorescent and DAPI overlapped images (Figure 3A–F). The immature neurons were characterized by positive MAP2 staining and either the absence of dendrites and axons or shorter dendrites (Figure 3A–F). Non-neural cells were also observed as only DAPI stained nucleus (Figure 3A–F). In the presence of both 50 µM and 100 µM of either BPA or BPF, there was a significant increase in the number of immature neurons relative to mature neurons (Figure 3B,C,E,F). Both the mature and immature neurons were enumerated and compared (Section 3.6).

### 3.4. Effects of BPA or BPF on rNSC Differentiation Directed into Oligodendrocytes

**Figure 4:**
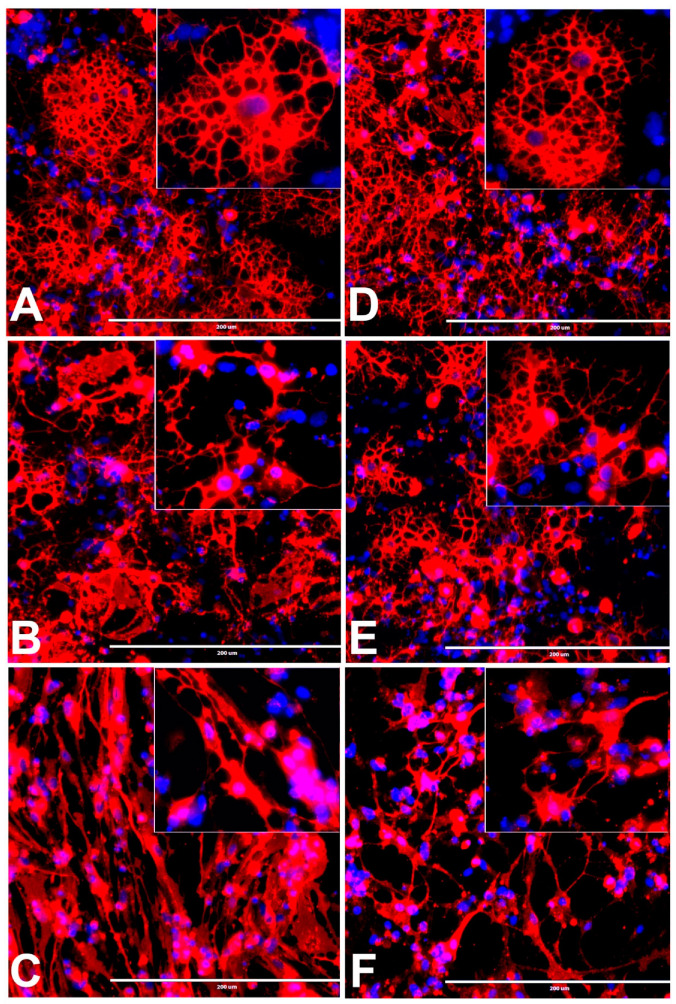
Effects of different doses of BPA or BPF on the morphological differentiation of rNSCs into oligodendrocytes. The rNSC cultured in oligodendrocyte directed differentiation media in the presence or absence of BPA or BPF for 7 days. Differentiated oligodendrocytes were visualized with oligodendrocyte-specific protein-marker mGalC and nuclei specific-marker DAPI. The images shown are representative fluorescent images of oligodendrocyte differentiation (**A**) in the absence of BPA (control), (**B**) in the presence of 50 μM BPA, (**C**) 100 μM BPA; (**D**) in the absence of BPF (control), (**E**) in the presence of 50 μM BPF, and (**F**) 100 μM BPF. Scale bar indicates 200 μm at 20× magnification.

**Figure 5:**
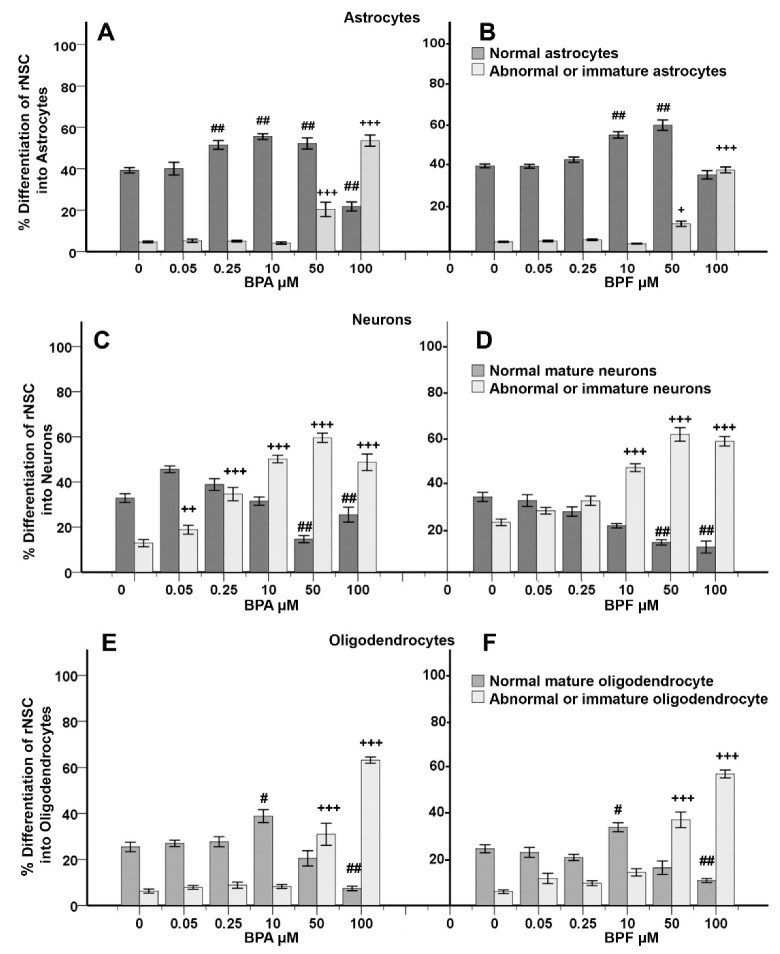
Effects of different concentrations of Bisphenol A (BPA) or Bisphenol F (BPF) treated for seven days, on the %differentiation of rNSC in the relevant cell-specific directed-differentiation medium. The %differentiation was enumerated as an index of the differentiation process of rNSCs into astrocytes (**A**,**B**), neurons (**C**,**D**), and oligodendrocytes (**E**,**F**), respectively. Control groups cells were differentiated into relevant cell types in the absence of BPA or BPF (0 µM). Differentiated astrocytes neurons and oligodendrocytes were visualized with cell-specific protein-markers GFAP, MAP2, and mGalc, respectively. Fluorescent, DAPI, and phase-contrast superimposed images were used for the quantification of the normal mature cell, and immature or abnormal cell types of each cell category. Each data point represents mean ± SE; ^+^ *p* < 0.05, ^++, #^ *p* < 0.01, ^+++, ##^ *p* < 0.001 compared with the relevant controls.

The rNSCs cultured with oligodendrocyte directed-differentiating medium were differentiated into immature and mature oligodendrocytes, with mature oligodendrocytes being more predominant in control cells (Figure 4A,D). Morphological differences were observed by phase-contrast microscopy, mGalc immunofluorescent, and DAPI overlapped images (Figure 4A–F). Mature oligodendrocytes characterized by a cell body with a nucleus and extended cytoplasmic processes that create highly branched network connections with other cells. Immature oligodendrocytes characterized by either shorter cellular process or only soma with no processes. These were similarly stained with specific marker mGalC (Figure 4A–F). Non-oligodendrocyte cells were also observed as blue DAPI stained nucleus without mGalC stained soma. In the presence of 50 and 100 µM BPA or BPF, rNSCs differentiated with increasing immature vs. mature oligodendrocytes in a concentration-dependent manner (Figure 4B,C,E,F). Both the mature and immature oligodendrocytes were enumerated and compared (Figure 5E,F).

### 3.5. Concentration-Response Effects of BPA and BPF on the Degree of % Differentiation of rNSCs Directed into Astrocytes

Welch’s ANOVA of individual concentration-response of BPA and BPF on “% differentiation” of rNSCs into mature astrocytes showed significant effects (Welch’s F(11, 46.22) = 27.92, *p* < 0.001). Multiple comparisons of the concentration-response data by Games–Howell post hoc test showed a significant increase in “% differentiation” of rNSCs into mature astrocytes in 0.25 μM (12.27 ± 2.46), 10 μM (16.29 ± 1.88), 50 μM (13 ± 3.00) BPA treated groups, and 10 μM (14.96 ± 1.79), 50 μM (19.67 ± 2.68) BPF treated groups compared with the relevant controls (Figure 5A,B). However, 100 μM BPA (17.46 ± 2.55%, *p* < 0.001) significantly decreased the % differentiation of rNSCs into mature astrocytes while 100 μM BPF (4.31 ± 2.20%) showed no significant difference compared with the relevant controls (Figure 5A,B).

Welch’s ANOVA of individual concentration-response of BPA and BPF on “% differentiation” of rNSCs into immature or abnormal astrocytes showed significant effects (Welch’s F(11, 46.03)= 81.64, *p* < 0.001). Multiple comparisons of the concentration-response data by Games–Howell post hoc test showed a significant increase in “% differentiation” of rNSCs into immature or abnormal astrocytes. The BPA treated groups significantly increased the % differentiation of rNSCs into immature or abnormal astrocytes at the concentrations of 50 μM (15.72 ± 3.53%) and 100 μM (48.94 ± 2.72%) BPA and 50 μM (8.79 ± 1.33%) and 100 μM (34.78 ± 1.46%) BPF treated groups compared with the relevant control groups.

**Figure 6:**
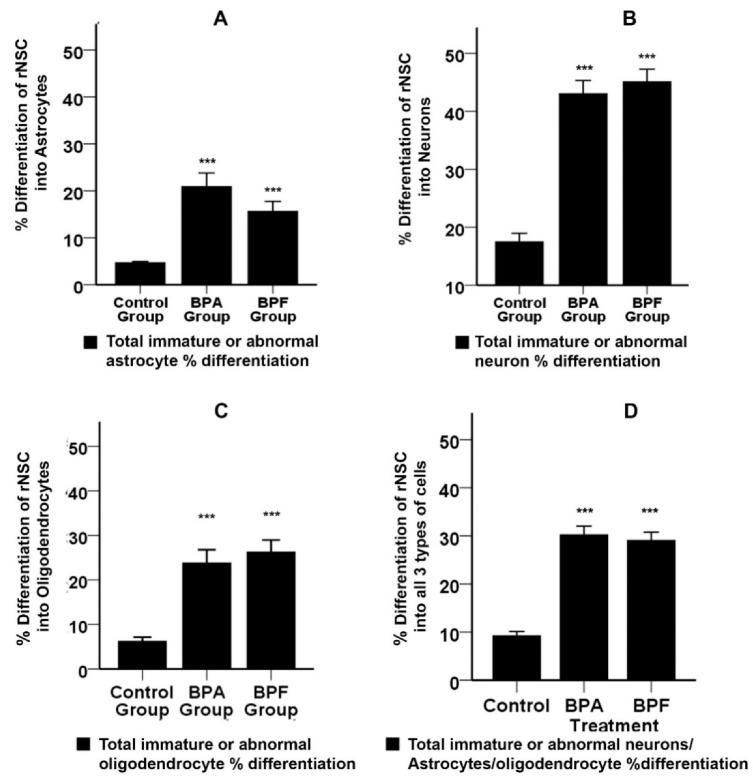
Effects of BPA or BPF on percentage differentiation of rNSCs into total immature or abnormal astrocytes, neurons, and oligodendrocytes as whole groups (pooled concentration-response-data). The %differentiation was enumerated as an index of the differentiation process of rNSCs into immature or abnormal (**A**) astrocyte, (**B**) neurons, and (**C**) oligodendrocytes (**D**) overall effects of BPA or BPF on all 3-types of cells (pooled immature or abnormal astrocytes, neurons, oligodendrocytes), respectively. Differentiated astrocytes, neurons, and oligodendrocytes visualized by cell-specific protein-marker GFAP, MAP2, and mGalc, respectively. Morphology with neurites in fluorescent, DAPI, and phase contrast superimposed images were used to quantify the immature or abnormal cells of each cell category. Each data point represents mean ± SE; *** *p* < 0.001 compared with the relevant control groups.

Group-wise analysis of total immature or abnormal astrocytes by Welch’s ANOVA showed that BPA and BPF have a significant effect on “% differentiation” of rNSCs into total immature or abnormal astrocytes (Welch’s F(2, 73.14) = 28.41, *p* < 0.001). Group-wise comparison by Games–Howell post hoc test showed a significant increase in the “% differentiation” of rNSCs into total immature or abnormal astrocytes in BPA treated group (16.21 ± 2.88%, *p* < 0.001) and BPF treated group (10.93 ± 2.13%, *p* < 0.001) when compared with the control group (Figure 6A).

### 3.6. Concentration-Response Effects of BPA and BPF on the % Differentiation of rNSCs Directed into Neurons

Welch’s ANOVA of individual concentration-response of BPA and BPF on “% differentiation” of rNSCs into mature neurons showed significant effects (Welch’s F(11, 45.25) = 37.266, *p* < 0.001). Multiple comparisons of the concentration-response data by Games–Howell post hoc test revealed a significant decrease in “% differentiation” of rNSCs into mature neurons. The BPA treated groups significantly decreased the % differentiation (*p* < 0.001) of rNSCs into mature neurons at the concentrations of 50 μM (18.18 ± 2.47%) and 100 μM (7.37 ± 3.83%) compared with the relevant control in a concentration-dependent manner (Figure 5C). Similarly, BPF treated groups significantly decreased the % differentiation (*p* < 0.001) of rNSCs into mature neurons at the concentrations of 50 μM (20.15 ± 2.34%) and 100 μM (22.15 ± 3.33%) compared with the relevant control group (Figure 5D). However, there is no significant effect of 0.05 μM, 0.25 μM, and 10 μM of both BPA and BPF on the “% differentiation” of rNSCs into mature neurons compared with the relevant controls (Figure 5C,D).

Welch’s ANOVA of individual concentration-response of BPA and BPF on “% differentiation” of rNSCs into immature neurons showed significant effects (Welch’s F(11, 445.237) = 67.032, *p* < 0.001). Multiple comparisons of the concentration-response data by Games–Howell post hoc test revealed a significant increase in “% differentiation” of rNSCs into immature neurons. The BPA treated groups significantly increased the % differentiation (*p* < 0.001) of rNSCs into immature neurons at the concentrations of 0.25 μM (21.74 ± 3.35%), 10 μM (37.27 ± 2.32%), 50 μM (46.70 ± 2.60%), and 100 μM (35.86 ± 3.98%) compared with the relevant control in a concentration-dependent manner (Figure 5C). Similarly, BPF treated groups significantly increased the “% differentiation” (*p* < 0.001) of rNSCs into immature neurons at the concentrations of 10 μM (24.17 ± 2.25%), 50 μM (38.92 ± 3.34%), and 100 μM (35.91 ± 2.53%) compared with the relevant control group (Figure 5D). There is no significant effect of individual concentration of 0.05 μM and 0.25 μM of BPF on the “% differentiation” of rNSCs into immature neurons compared with the relevant controls (Figure 5D), whereas all the concentrations of BPA used showed a significant increase in “% differentiation” of rNSCs into immature neurons (Figure 5C) compared with the relevant control, indicating that BPA has more effect compared with BPF. Group-wise analysis of immature or abnormal neurons by Welch’s ANOVA showed that BPA and BPF have a significant effect on “% differentiation” of rNSCs into total immature or abnormal neurons (Welch’s F(2, 81.129) = 78.66, *p* < 0.001). Group-wise comparison by Games–Howell post hoc test revealed a significant increase in the “% differentiation” of rNSCs into total immature or abnormal neurons in BPA treated group (25.56 ± 2.66%, *p* < 0.001,) and BPF treated group (27.61 ± 2.55%, *p* < 0.001) when compared with the control group (Figure 6B).

### 3.7. Concentration-Response Effects of BPA and BPF on the % Differentiation of rNSCs into Oligodendrocytes

Welch’s ANOVA test showed that BPA and BPF have a significant effect on “% differentiation” of rNSCs into mature oligodendrocytes (Welch’s F(11, 46.68) = 32.35, *p* < 0.001). Multiple comparisons of the concentration-response data by Games–Howell post hoc test revealed a significant increase in “% differentiation” of rNSCs into mature oligodendrocytes at 10 μM of BPA (13.42 ± 3.42%) and 10 μM of BPF (9.34 ± 2.60%) compared with the relevant control, whereas 50 μM BPA (4.95 ± 3.92%) and 50 μM BPF (8.25 ± 3.40%) did not reduce the “% differentiation” of rNSCs into mature oligodendrocytes. However, 100 μM BPA (18.01 ± 2.30%) and 100 μM BPF (13.78 ± 1.92%) significantly reduced the % differentiation of rNSCs into mature oligodendrocytes (Figure 5E,F).

Data analysis by Welch’s ANOVA test showed that BPA and BPF have a significant effect on “% differentiation” of rNSCs into immature oligodendrocytes (Welch’s F(11, 47.22) = 187.74, *p* < 0.001). Multiple comparisons of the concentration-response data by Games–Howell post hoc test revealed a significant increase in “% differentiation” of rNSCs into immature oligodendrocytes. The BPA treated groups significantly increased the “% differentiation” of rNSCs into immature oligodendrocytes at the concentrations of 50 μM (24.65 ± 4.87%, *p* < 0.01) and 100 μM (56.91 ± 1.60%, *p* < 0.001) relative to control, in a concentration-dependent manner (Figure 5E). Similarly, BPF treated groups significantly increased the “% differentiation” (*p* < 0.001) of rNSCs into immature oligodendrocytes at the concentrations of 50 μM (31.24 ± 3.46%) and 100 μM (51.12 ± 1.86%) compared to the relevant control group (Figure 5F). There is no significant effect on the “% differentiation” of rNSCs into immature oligodendrocytes in the lower dose concentrations (0.05 μM, 0.25 μM, and 10 μM) in either BPA or BPF treatment (Figure 5E,F). Group-wise analysis of total immature or abnormal oligodendrocytes by Welch’s ANOVA showed that BPA and BPF have a significant effect on “% differentiation” of rNSCs into immature oligodendrocytes (Welch’s F(2, 77.66) = 45.58, *p* < 0.001). Further, group-wise comparison by Games–Howell post hoc test revealed a significant increase in the “% differentiation” of rNSCs into total immature or abnormal oligodendrocytes in BPA treated group (21.25 ± 3.38 %, *p* < 0.001) and BPF treated group (20.18 ± 2.70 %, *p* < 0.001) when compared with the control group (Figure 6C).

Furthermore, a comparison of overall effects of BPA or BPF treatments on immature or abnormal cells in all 3-types of cells (astrocytes, neurons, oligodendrocytes) by Games–Howell post hoc test showed a significant increase in %differentiation in BPA treated group (20.98 ± 1.96, *p* < 0.001) and BPF treated (19.83 ± 1.85, *p* < 0.001) group (Figure 6D).

### 3.8. Effects of BPA or BPF on Cell Proliferation of Differentiating rNSCs into Astrocytes

**Figure 7:**
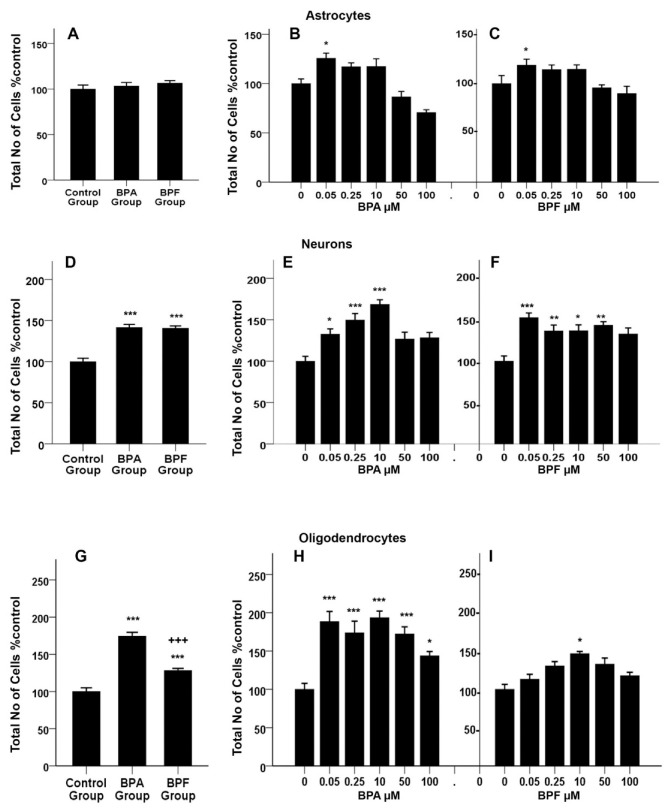
Effects of different concentrations of Bisphenol A (BPA) or Bisphenol F (BPF) treatment for seven days on cell proliferation of differentiating rNSCs into astrocytes, neurons, and oligodendrocytes. The “total number of cells % control” was enumerated as an index of cell proliferation in, (**B**,**C**) astrocytes, (**E**,**F**) neurons, and (**H**,**I**) oligodendrocytes, respectively. Group-wise comparisons of BPA and BPF for astrocytes (**A**), neurons (**D**), and oligodendrocytes (**G**) are illustrated. Differentiated astrocytes, neurons, and oligodendrocytes were stained with specific protein-markers GFAP, MAP2-clone-AP18, and mGalc, respectively. DAPI stained nuclei of fluorescent and phase-contrast superimposed images were enumerated as “the total number of cells %control” of each cell category. “Total No of Cells %control” = (No of total nuclei of treated cells ÷No of total nuclei of control cells) × 100. Each data point represent mean ± SE; * *p* < 0.05, ** *p* < 0.01, ***, ^+++^ *p* < 0.001 compared with the relevant control groups.

The “total number of cells % control” was estimated as an index of cell proliferation or the cytotoxicity of differentiating rNSCs into astrocytes, neurons, and oligodendrocytes throughout the study. A two-way ANOVA was conducted to examine the effect of BPA and BPF dose on cell proliferation or cytotoxicity in these cells. In differentiating astrocytes, there was a statistically significant effect of BPA and BPF on the “total number of cells % control”, (F(9, 117) = 9.901, *p* < 0.01, η2p = 0.432, observed power = 1). Multiple comparisons of the concentration-response of BPA and BPF by Bonferroni post hoc test revealed that there is a statistically significant difference in cell proliferation at 0.05 μM of both BPA and BPF treated cells. Statistically significant differences were not observed in the ‘total number of cells % control” at 0.25 μM, 10 μM, and 50 μM for both BPA (Figure 7B) and BPF (Figure 7C) treated groups. However, group-wise comparison by Bonferroni post hoc test revealed no significant change in the “total number of cells % control” of rNSCs into total astrocytes in either BPA or BPF treated group when compared with the control group (Figure 7A).

### 3.9. Effects of BPA or BPF on Cell Proliferation of Differentiating rNSCs into Neurons

Two-way ANOVA showed statistically significant effect of BPA and BPF on “total number of cells % control” (F(9, 116) = 4.173, *p* < 0.01, η2p = 0.245, observed power = 0.889) of differentiating neurons. Multiple comparisons of the dose–response of BPA by Bonferroni post hoc test showed that there are significant increases in the “total number of cells % control” at 0.05 μM (32.62 ± 8.86 *p* < 0.05), 0.25 μM (49.59 ± 9.28, *p* < 0.001), and 10 μM (68.67± 8.86, *p* < 0.001) in comparison with the relevant control group. However, 50 μM and 100 μM of BPA did not show any significant differences compared to the relevant control (Figure 7E). Similarly, BPF treated cells revealed a significant increase in the “total number of cells % control” at 0.05 μM (52.57 ± 9.28 *p* < 0.001), 0.25 μM (36.44 ± 9.05, *p* < 0.01), 10 μM (36.71 ± 9.54, *p* < 0.05), and 50 μM (43.40 ± 8.86, *p* < 0.01), while 100 μM of BPF treatment did not show a significant difference (Figure 7F). Furthermore, group-wise comparison by Bonferroni post hoc test revealed a significant increase in the ‘total number of cells % control” of rNSCs into total neurons in both BPA (41.59 ± 5.35%, *p* < 0.001) and BPF (40.70 ± 5.41%, *p* < 0.001) treated group compared to the control group (Figure 7D).

### 3.10. Effects of BPA or BPF on Cell-Proliferation of Differentiating rNSCs into Oligodendrocytes

There is a statistically significant effect of BPA and BPF on “total number of cells % control” (F(9, 122) = 3.75, *p* < 0.001, η2p = 0.217, observed power = 0.832) of differentiating oligodendrocytes as determined by two-way ANOVA. Multiple comparisons of the concentration-response of BPA by Bonferroni post hoc test showed that all concentrations of BPA significantly increase the “total number of cells % control” of differentiating oligodendrocytes. In brief, significant increase in the “total number of cells % control” were seen at 0.05 μM (88.39 ± 12.41 *p* < 0.001), 0.25 μM (73.80 ± 12.12, *p* < 0.001), 10 μM (93.52 ± 11.67, *p* < 0.001), 50 μM (72.39 ± 12.41, *p* < 0.001), and 100 μM (43.70 ± 11.88, *p* < 0.05) compared to relevant control group (Figure 7H). In contrast, BPF treated cells only showed a significant increase in the “total number of cells % control” at 10 μM (36.71 ± 9.54, *p* < 0.05), while all other concentrations of BPF treated cells did not show a significant difference (Figure 7I). Group-wise comparison showed a statistically significant increase in the “total number of cells % control” of both BPA (74.28 ± 7.22, *p* < 0.001) and BPF (28.07 ± 7.19, *p* < 0.001) treated groups compared with relevant controls (Figure 7G). Similarly, there is a significant increase in the “total number of cells % control” of the BPA treated group compared (46.21 ± 5.19, *p* < 0.001) with the BPF treated group (Figure 7G).

### 3.11. Effects of BPA or BPF on the Morphologies of Astrocytes, Oligodendrocytes, and Neurons Differentiated from rNSCs

**Figure 8:**
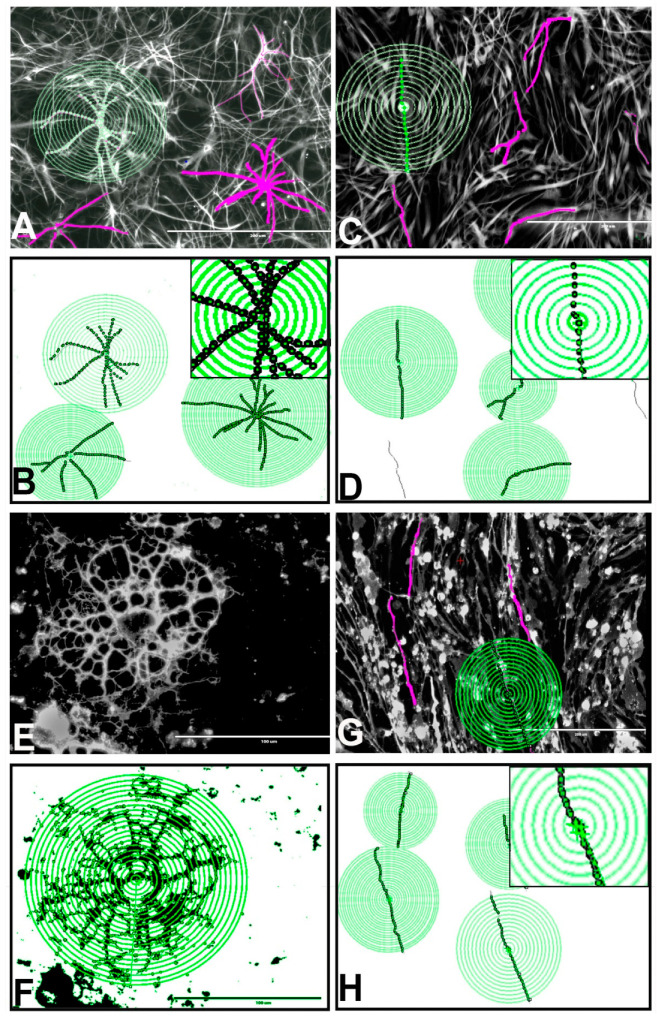
Effects of BPA or BPF treated for seven days on morphological changes of astrocytes and oligodendrocytes differentiated from rNSCs in relevant cell-specific directed differentiation medium. Differentiated astrocytes and oligodendrocytes were visualized with specific protein-marker GFAP and mGalc, respectively, and nuclei stained with DAPI. Fluorescent signals were segmented using the threshold tool and converted to binary mask for tracing complete cell morphology. Representative overlapped image of the rNSC differentiated into astrocytes in the absence of BPF (**A**—control) and the presence of 100 µM BPF (**C**). Relevant “Sholl analysis binary images” of traced astrocytes in the absence of BPF (**B**—control) and the presence of 100 µM BPF (**D**). Traced astrocytes with “simple neurite tracer” are in pink. Representative image of the rNSC differentiated into oligodendrocytes in the absence of BPA (**E**—control) and the presence of 100 µM BPA (**G**). Relevant “Sholl analysis binary images” of traced oligodendrocytes in the absence of BPA (**F**—control) and the presence of 100 µM BPA (**H**). Scale bar indicates 200 μm at 20× magnification.

BPA and BPF reduced the complexity of cellular process arbors and changed the morphology of astrocytes, oligodendrocytes, and neurons differentiated from rNSCs. Traced images of complete astrocytes in pink color (Figure 8A,C) oligodendrocytes (Figure 8E,G), and neurons are converted into binary files and created “Sholl circle” to measure the number of intersections per each “Sholl circle” for astrocytes (Figure 8B,D), oligodendrocytes (Figure 8F,H), and neurons. 100 µM BPF (Figure 8C,D) reduced the number of astrocyte processes and changed the astrocyte morphology compared with the control (Figure 8A,B). 100 µM and 50 µM of BPA and 50 µM of BPF as well showed similar effects (data not shown). Furthermore, 100 µM of BPA (Figure 8G,H) reduced the number of oligodendrocyte processes and changed the oligodendrocyte morphology to linearly elongated oligodendrocytes compared to the control oligodendrocytes with characteristic highly branched complex morphology (Figure 8E,F). Additionally, 100 µM and 50 µM BPF and 50 µM BPA showed similar effects (data not shown). These morphological changes were further quantified by Sholl analysis [47,69].

### 3.12. Effects of BPA and BPF on Sholl Analysis on Astrocytes

**Figure 9:**
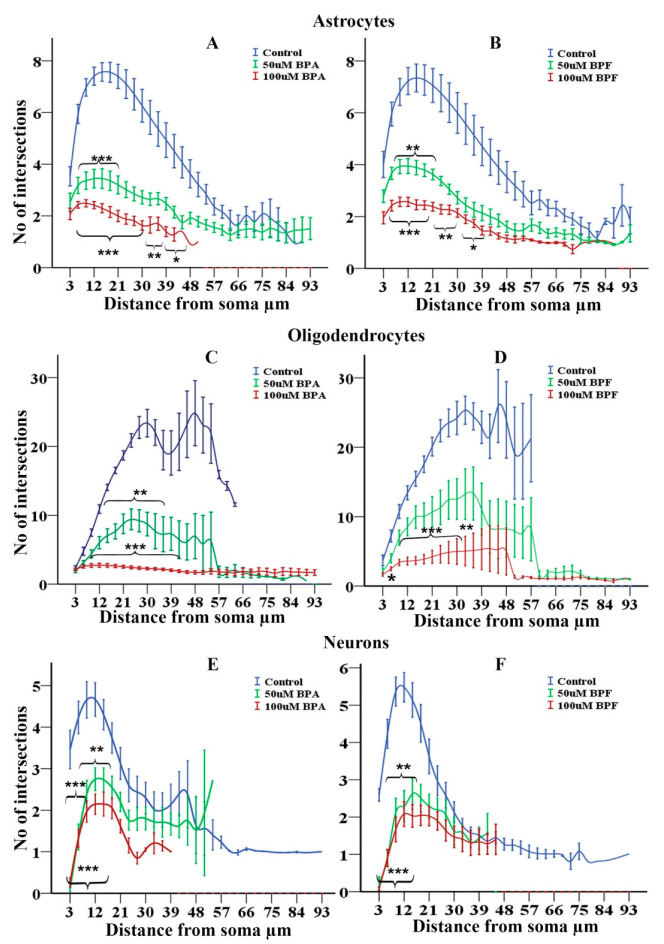
Effects of different doses of BPA or BPF treatment for seven days on morphometric analysis of astrocytes, oligodendrocytes, and neurons differentiated from rNSCs in relevant cell-specific directed differentiation medium. The number of intersections between the cellular processes and “Sholl circle” was used as an index of morphological changes of astrocytes differentiated from rNSCs (**A**,**B**), oligodendrocytes (**C**,**D**), and neurons (**E**,**F**). Differentiated astrocytes (*n* = 216–222 per group), oligodendrocytes (*n* = 178–180 per group), and neurons (*n* = 271–261 per group) were stained with specific markers GFAP, mGalc, and MAP2, respectively, and nuclei stained with DAPI. Fluorescent signals were segmented and transformed into a binary mask for tracing complete cell morphology. Traced cell images were subjected to morphometric Sholl analysis to enumerate a number of intersection in each “Sholl circle”. Multiple comparisons of the number of intersections within 3 μm to 48 μm regions of astrocytes, oligodendrocytes, and neurons differentiated in the presence and absence of BPA or BPF are illustrated. Data shown are mean ± SE; * *p* < 0.05, ** *p* < 0.01, *** *p* < 0.001 compared with the relevant control groups.

Sholl analysis [47,69] of astrocytes differentiated from rNSCs in the presence of BPA revealed a statistically significant effect of BPA on the mean intersections of astrocyte processes in “Sholl circles” (F(2, 602) = 315.31, *p* < 0.001, η2p = 0.512, observed power = 1.00) as determined by two-way ANOVA. Multiple comparisons of all groups by Games–Howell post hoc test showed a statistically significant reduction in the mean intersections of astrocyte processes in “Sholl circles” in 50 μM (2.82 ± 0.11, *p* < 0.001) and 100 μM BPA treated group (2.08 ± 0.12, *p* < 0.001) in comparison with the control group (5.85 ± 0.10). Individual comparison of the number of intersections per 3 μm distance by Games–Howell post hoc test revealed that there is a significant reduction of the number of intersections within 9 μm to 48 μm radius from the soma of the 100 μM BPA treated astrocytes compared with the corresponding radius (9 μm to 48 μm) of the control astrocytes (Figure 9A). Meanwhile, 50 μM BPA treated astrocyte group also reduced the number of intersections within 9 μm to 21 μm radius from the soma compared with the corresponding radius (9 μm to 21 μm) of the control astrocytes (Figure 9A).

Similarly, astrocytes differentiated from rNSCs in the presence of BPF also revealed a statistically significant effect of BPF on the mean intersections of astrocyte processes in “Sholl circles” (F(2, 741) = 310.7, *p* < 0.001, η2p = 0.456, observed power = 1.00) as determined by two-way ANOVA. Multiple comparisons of the three groups by Games–Howell post hoc test showed a statistically significant reduction in the mean intersections of astrocyte processes in “Sholl circles” of 50 μM BPF treated group (3.006 ± 0.103, *p* < 0.01) and 100 μM BPF treated group (2.07 ± 0.09, *p* < 0.001) in comparison with the control group (5.74 ± 0.11). Individual comparison of a number of the intersection per 3 μm distance by Games–Howell post hoc test revealed that there is a significant reduction of the number of intersections in BPF treated astrocytes compared with the control astrocytes. Further, 100 μM BPF treated cells showed a significant reduction of the number of intersection between 9 μm to 39 μm radius from the soma of the astrocytes intersection compared with the corresponding radius of the control astrocytes (Figure 7B,D and Figure 9B). Meanwhile, 50 μM BPF treated astrocytes reduced the number of intersections to a lesser extent within 9 μm to 21 μm radius from the soma compared with the control astrocytes (Figure 9B).

**Figure 10:**
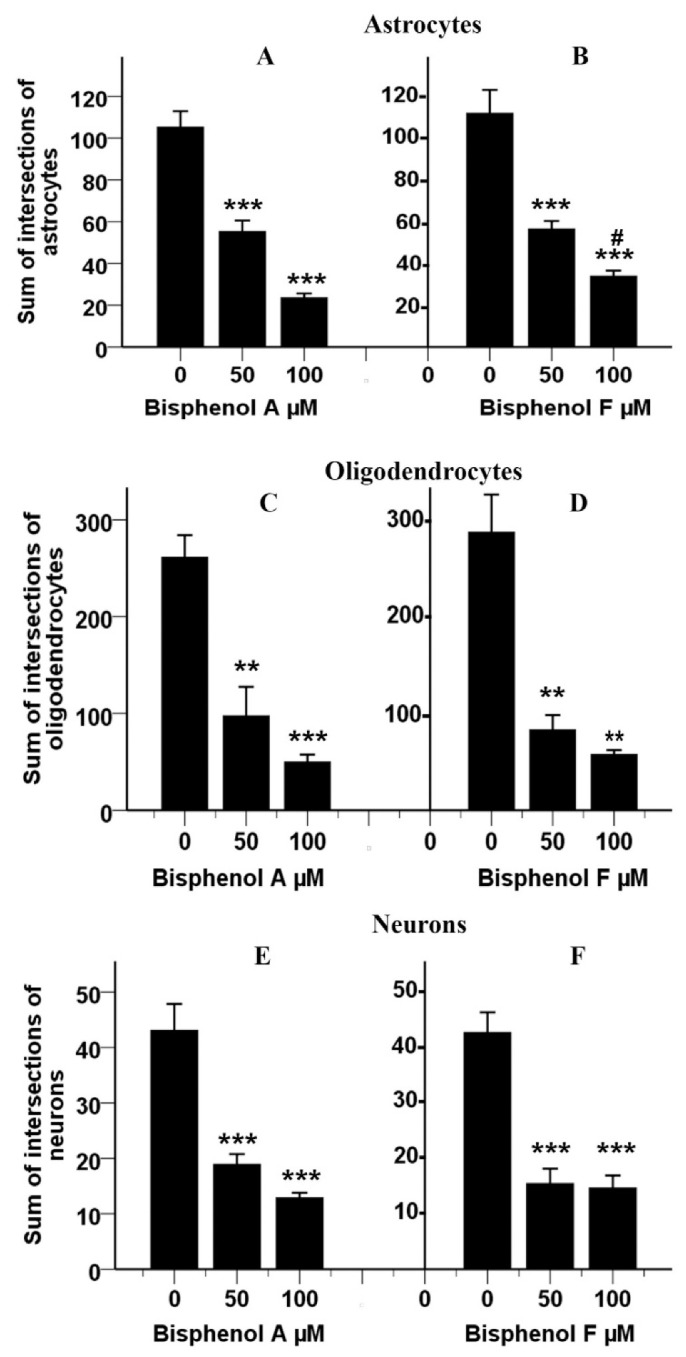
Effects of different concentrations of BPA or BPF treated for seven days on morphometric analysis of astrocytes, oligodendrocytes, and neurons differentiated from rNSCs in relevant cell-specific directed differentiation medium. The sum of intersections of cellular processes per cell was used as an index of morphological changes of astrocytes (**A**,**B**), oligodendrocytes (**C**,**D**), and neurons (**E**,**F**). Differentiated astrocytes (*n* = 15–19 per group), oligodendrocytes (*n* = 11–18 per group), and neurons (*n* = 18–29 per group) were visualized with specific protein-marker GFAP, mGalc, and MAP2, respectively, and nuclei stained with DAPI. Fluorescent signals were segmented and transformed to binary mask for tracing complete cell morphology. Traced cell images were subjected to Sholl analysis to enumerate the sum of intersections per cell. Each data points represents mean ± SE; ** *p* < 0.01, *** *p* < 0.001 compared with the relevant control groups. # *p* < 0.05 compared with 100 μM BPA treated group.

To confirm these findings, we measured the mean value of the sum of intersections for each cell type. Astrocytes differentiated from rNSCs in the presence of BPA or BPF showed a statistically significant effect (F(5, 88) = 38.66, *p* < 0.001, η2p = 0.687, observed power = 1.00) on the sum of intersections per astrocyte as determined by ANOVA. Absolute values for the sum of intersections per each cell type are depicted in Figure 10A,B. Games–Howell post hoc test showed a statistically significant reduction in the sum of intersections in 100 μM BPA treated astrocytes (81.67 ± 7.74, *p* < 0.001) and 50 μM BPA treated astrocytes (49.95 ± 9.09, *p* < 0.001) compared to control (Figure 10A). Similarly, 100 μM BPF treated astrocytes (77.89 ± 11.44, *p* < 0.001) and 50 μM BPF treated astrocytes (55.36 ± 11.76, *p* < 0.01) also revealed a statistically significant reduction in the sum of intersections compared with relevant control (Figure 10B). Further, 100 μM BPF treated astrocytes showed a significant increase in the sum of intersections (10.36 ± 3.16, *p* < 0.05) compared with the 100 μM BPA treated astrocytes (Figure 10A,B).

### 3.13. Effects of BPA and BPF on Sholl Analysis on Oligodendrocytes

Sholl analysis [47,69] of oligodendrocytes differentiated from rNSC in the presence of BPA revealed a statistically significant effect of BPA on the mean intersections of processes in “Sholl circles” (F(2, 595) = 319.86, *p* < 0.001, η2p = 0.518, observed power = 1.00) as determined by two-way ANOVA. Group-wise multiple comparisons by Games–Howell post hoc test revealed a statistically significant reduction in the mean intersections of processes in “Sholl circles” in 50μM BPA treated group (6.67 ± 0.38, *p* < 0.001) and 100 μM BPA treated group (2.30 ± 0.33, *p* < 0.001) compared to control (16.32 ± 0.44). Individual comparison of the number of intersections per 3 μm distance by Games–Howell post hoc test revealed that there is a significant reduction of the number of intersections in BPA treated oligodendrocytes compared with the control oligodendrocytes. Further, 100 μM BPA treated oligodendrocytes showed a significant reduction of the number of intersection within 9 μm to 42 μm radius from the soma as compared to the corresponding radius of the control oligodendrocytes (Figure 8G,H and Figure 9C). Meanwhile, 50 μM BPA treated oligodendrocytes reduced the number of intersections within 9 μm to 36 μm radius from the soma compared with the corresponding radius from the soma of the control oligodendrocytes (Figure 9C).

Similarly, there is a statistically significant effect of BPF on the mean intersections of processes in “Sholl circles” (F(2, 502) = 147.23, *p* < 0.001, η2p = 0.370, observed power = 1.00) of oligodendrocytes differentiated from rNSCs in the presence of BPF. Multiple comparisons of the three groups by Games–Howell post hoc test revealed a statistically significant reduction in the mean intersections of processes in “Sholl circles” in 100 μM BPF treated group (4.19 ± 0.569, *p* < 0.001) compared with the control group (17.85 ± 0.566). Individual comparison of the number of intersections per 3 μm distance by Games–Howell post hoc test revealed that there is a significant reduction of the number of intersections within a 6 μm to 33 μm radius from the soma of the 100 μM BPF treated oligodendrocytes compared with the control oligodendrocytes (Figure 9D). However, 50 μM BPF did not significantly reduce the number of intersections radiating from the soma of oligodendrocytes (Figure 9D).

Further confirmation of these morphometric analyses of oligodendrocytes was established by measuring the mean value of the sum of intersections of an oligodendrocyte. Oligodendrocytes differentiated from rNSCs in the presence of BPA or BPF also showed a statistically significant effect (F(5,70) = 23.28, *p* < 0.001, η2p = 0.625, observed power = 1.00) on the sum of intersections per oligodendrocyte as determined by ANOVA. Absolute values for the sum of intersections per oligodendrocyte are depicted in Figure 10C,D. Statistically significant reductions were observed on the sum of intersections in 100 μM BPA treated oligodendrocytes (211.33 ± 24.10, *p* < 0.001) and 50 μM BPA treated oligodendrocytes (163.70 ± 37.58, *p* < 0.01) compared with the relevant control group (Figure 10C) as determined by Games–Howell post hoc test. Similarly, 100 μM BPF treated oligodendrocytes (229.48 ± 39.01, *p* < 0.01) and 50 μM BPF treated oligodendrocytes (204.09 ± 41.58, *p* < 0.01) also showed a statistically significant reduction in the sum of intersections compared with relevant control oligodendrocytes (Figure 10D).

### 3.14. Effects of BPA and BPF on Sholl Analysis on Neurons

Sholl analysis [47,69] of neurons differentiated from rNSCs in the presence of BPA revealed a statistically significant effect of BPA on the mean intersections in “Sholl circles” of neural dendrites (F(2, 630) = 74.26, *p* < 0.001, η2p = 0.191, observed power = 1.00) as determined by two-way ANOVA. Multiple comparisons of the three groups by Games-Howell post hoc test showed a statistically significant reduction in the mean intersections in “Sholl circles” of neural dendrites of 50μM BPA treated neurons (1.19 ± 0.09, *p* < 0.001) and 100 μM BPA treated neurons (1.54 ± 0.10, *p* < 0.001) compared with the control neurons (3.15 ± 0.09). Individual comparison of the number of intersections per 3 μm distance by Games–Howell post hoc test revealed that there is a significant reduction of the number of intersections within 3 μm to 18 μm radius from the soma of the 100 μM and 50 μM BPA treated group compared with the corresponding radius of the control group (Figure 9E).

Similarly, neurons differentiated from rNSCs in the presence of BPF also showed a statistically significant effect of BPF on mean intersections in “Sholl circles” of neuron dendrites (F(2, 790) = 58.98, *p* < 0.001, η2p = 0.130, observed power = 1.00) as determined by two-way ANOVA. Multiple comparisons of the three groups by Games–Howell post hoc test showed a statistically significant reduction in the mean intersections in 50μM (1.168 ± 0.11, *p* < 0.01) and 100 μM BPF treated group (1.53 ± 0.10, *p* < 0.001) compared with the control group (3.16 ± 0.10). Individual comparison of the number of intersections per distance by Games–Howell post hoc test revealed that there is a significant reduction of the number of intersections within 3 μm to 15 μm radius from the soma of the neurons in the 100 μM and 50 μM BPF treated group compared with the corresponding radius of the control group (Figure 9F).

Confirmations of these morphometric analyses of neurons were established by measuring the mean value of the sum of intersections of neurons. BPA and BPF showed a statistically significant effect (F(5, 136) = 25.82, *p* < 0.001, η2p = 0.487, observed power = 1.00) on the sum of intersections in neurons differentiated from rNSCs as determined by ANOVA. Games-Howell post hoc test revealed a statistically significant reduction in the sum of intersections in 100 μM BPA treated neurons (30.20 ± 4.83, *p* < 0.001) and 50 μM BPA treated neurons (24.13 ± 5.07, *p* < 0.001) compared with the relevant control group (Figure 10E). Similarly, 100 μM BPF treated neurons (28.05 ± 4.21, *p* < 0.001) and 50 μM BPF treated neurons (27.27 ± 4.45, *p* < 0.001) also showed a statistically significant reduction in the sum of intersections compared with relevant control neurons (Figure 10F). Further, no significant differences were observed between BPA and BPF treated neurons (Figure 10E,F).

## 4. Discussion

Currently, there are limited data on the developmental effects of the BPA analogues, hence this study was designed to compare and evaluate the neurodevelopmental effects of BPA and BPF on rNSCs. We used a monolayer based assay model [46] to direct rNSCs differentiation into astrocytes, neurons, and oligodendrocytes in the presence or absence of BPA or BPF. It is established facts that recombinant human FGFb and EGF (20 ng/mL) prevent differentiation of rNSC, keep their stem cell properties, and proliferate rNSCs very rapidly in the NSC growth medium. In the presence of recombinant human FGFb and EGF rNSCs increase cell proliferation, but cannot initiate and proceed to the differentiation processes even BPA or BPF present in the rNSC growth medium. The %differentiation of rNSCs into astrocytes, neurons, and oligodendrocytes in the absence of BPA or BPF treatment (control groups) were approximately 40%, 49%, and 32%, respectively. In the presence of BPA, the percentage differentiation of rNSCs into astrocytes, neurons, and oligodendrocytes increased approximately to 61%, 74%, and 50%, respectively. Similarly, in the presence of BPF, the percentage differentiation increased approximately to 59%, 66%, and 48%, respectively. In support of these findings, male rats exposed to 400 μg/kg bw/day showed increased numbers of neurons and glia (astrocytes and oligodendrocytes) [75] while female rats exposed to 40 μg/kg bw/day of BPA showed an increased number of glial cells [76] in the medial prefrontal cortex.

Our results showed that exposure to 50 µM and 100 µM of either BPA or BPF lead to a significant reduction in cellular arborization and morphological changes for astrocytes, oligodendrocytes, and neurons. Sholl analysis of both BPA and BPF treated astrocytes showed a reduction in the number of intersections per “Sholl circle” within 9 μm to 48 μm radius from the soma for BPA and within 9 μm to 39 μm radius from the soma for BPF. Furthermore, both BPA and BPF significantly and comparably reduced the sum of intersections of astrocytes, reducing the complexity of cellular process arbors. Additionally, the treatment with 50 µM and 100 µM of BPA or BPF resulted in morphological changes in the astrocytes. The astrocytes differentiated from rNSC in the presence of 50 uM and 100 µM of BPA or BPF showed shorter or no processes and the four types of astrocytes were reduced or no longer visible. Astrocytes are involved in multiple functions, such as structural and metabolic support to neurons, maintaining synaptic connectivity, pruning circuit, regulating blood circulation, and the formation of the blood–brain barrier. Our findings correlate with the previous publication [77], which showed that BPA exposure to mouse astrocytes and neuron/glia co-cultures altered dopamine responsiveness in neurons and astrocytes.

Similarly, for oligodendrocytes, there are changes in cellular arborization in the presence of 50 µM and 100 µM of both BPA and BPF. BPA reduced the number of intersections per “Sholl circle” within 9 μm to 42 μm radius from the soma, whereas BPF reduced the number of intersections within 6 μm to 33 μm radius from the soma. However, further examination of the morphology of oligodendrocytes revealed that both BPA and BPF significantly reduced the sum of intersections, leading to a reduction in the complexity of cellular processes arbors. In addition to the changes in the arborization, there was a concentration-dependent reduction in the number of mature oligodendrocytes and an increase in immature oligodendrocytes. Mature oligodendrocytes were characterized by a cell body with a nucleus and extended cellular processes. Whereas the immature oligodendrocytes were characterized by either shorter cellular processes or soma with no cellular processes. Both were similarly stained with the specific marker mGalC. The morphological changes can affect the function of oligodendrocytes and neurons interactions. During central nervous system development, myelination of multiple axons of neurons requires the outgrowth and extensive branching of oligodendroglial processes [46]. To achieve this process, rNSC undergo a complex differentiation and maturation process, with different types of molecular and morphological changes. Subsequently, a number of long branched, and morphologically complex cell processes are elaborated [78]. Our data on morphological Sholl analysis showed that the high concentration of both BPA and BPF altered oligodendrocyte morphology and could impact differentiation and cellular function.

For neurons, Sholl analysis of 50 µM and100 µM BPA or BPF treated cells showed significantly reduced complexity of neuron dendrites arborization, required for circuit connectivity and brain functions. Significant reduction in the number of intersections per “Sholl circle” within 3 μm to 18 μm radius from the soma of BPA or BPF treated neurons, suggesting both BPA and BPF possess comparable adverse effects. Furthermore, both BPA and BPF significantly reduced the sum of intersections of neurons reducing the complexity of neuron dendrites arbors. At these doses, there were more immature than mature neurons. The mature neurons had different types of axonal morphologies, such as unipolar, bipolar, multipolar, and pyramidal like neurons, while the immature neurons had shorter or no dendrites and axons. Characteristic morphology of different types of neurons is very important for brain functions, circuit connectivity and regulations of multiple aspects of neural tissue homeostasis.

The effects on proliferation or cytotoxicity of both BPA and BPF treatment was examined on astrocytes, neurons, and oligodendrocytes. Multiple comparisons of the concentration-response on rNSCs differentiating into astrocytes showed a significant increase in “total number of cells % control” only at the 0.05 μM concentration and no effect on proliferation was seen at higher concentrations. There was no proliferation/cytotoxicity with either BPA or BPF treated groups overall, and these results are in agreement with the findings of Wise et al. [76]. According to these authors, BPA did not change the number of astrocytes in the rat prefrontal cortex of the investigated rats. Astrocytes, the major component of the glial cell population, contribute to a number of essential functions. These functions provide metabolic and physical functions to other neural cells and controlling synaptogenesis and myelination processes in the development of the brain.

Similar observations were made for neurons, multiple comparisons of the concentration-response of both BPA and BPF showed a significant increase in “total number of cells % control” indicating cell proliferation occurred at lower concentrations, but not at higher concentrations. Our findings are supported by previously published data, suggesting that a lower dose of BPA increase neural progenitor cell proliferation in vitro [79], while at higher concentrations, 200 μM and above for 24-h treatment, it decreases the cell proliferation indicating cytotoxicity [80,81,82]. Enhanced cell proliferation of neural stem cells/progenitor cells [79] in vitro by low concentrations of BPA may be due to functional and structural similarity of BPA to estradiol hormones, which also enhance cell proliferation. These findings are parallel to our finding in the rNSC differentiation process into neurons.

Additionally, group-wise comparison of both BPA and BPF revealed a significant increase in “total number of cells % control” of rNSCs differentiating into oligodendrocytes representing cell proliferation compared with the relevant controls.

Our data suggest that both BPA and BPF enhance the directed-differentiation process of rNSC into astrocytes, oligodendrocytes, neurons, and also interfere with the development of their characteristic morphology. These findings are in agreement with Verbanck et al. [83], as they showed that analysis of non-coding and coding RNA profile of adipocytes in the presence and absence of BPF or BPS interfere with genes and pathways responsible for hormones or hormone-like chemicals and have to be considered as endocrine disruptors like BPA [83]. Further, BPF, BPA, and its analogues (BPS and BPAF) induce in vivo expression of the cyp19a1b gene (cytochrome P450 or brain aromatase) in the developing brain of zebrafish during the early stages [14,84]. According to other studies, the cyp19a1b gene in the radial glial cells, serve as progenitors in the embryonic as well as adult neurogenesis [85,86,87]. Recently published data suggests that BPA, BPF, and their analogues can induce and/or repress certain genes in the hypothalamus, prefrontal cortex, and the medial preoptic area, and likely other brain areas [84,88,89]. Further, BPF, BPA, and their analogues induce hormonal imbalances in the synthesis and circulating levels of 17β estradiol, T3, and T4 hormone synthesis and circulating levels [14,89].

Recent comparative investigations using different animal models such as C. elegans, zebrafish, and rodents proved that BPF and BPA analogues have greater or equivalent neuroendocrine disruptive effects comparable to BPA [14,88,90,91,92]. Another study has shown that BPA, BPF, and BPS possess equivalent potential to activate human ESR1 and ESR2 [23,93], which are known to have a wide range of endocrine physiological networks [29] including upregulation of postsynaptic density protein, synaptophysin, and α-amino-3-hydroxy-5-methyl-4-isoxazole-propionic acid receptors expression in glutamatergic neurons [94]. A human post-mortem study using the hypothalamic and white matter of the brain from obese and normal weight individuals showed that low concentrations of BPF (2.2 ng/g) and BPA (1.2 ng/g) in the hypothalamus and BPF (2.3 ng/g), and BPA (1.0 ng/g) in white matter, were linked to their obese conditions [29]. The detection of BPA and BPF in the brain regions shows that they can cross the blood–brain barrier, which is predominantly composed of astrocytes, and consequently interfere with the endocrine physiological networks. Furthermore, several occupational exposure studies on BPA have reported higher concentrations of BPA in urine and serum [9,95,96,97]. In one occupational exposure study, BPA was detected at 101.94 µg/L in serum samples of 20 workers [9,98]. The BPA detected in the serum is equivalent to 0.447 µM BPA, which is similar to the amounts used in our study. Additionally, other studies on BPA levels on pregnant women reported that BPA levels in the blood plasma in the range of 0.5–22.3 ng/mL in the USA [99], and 0.3–18.9 ng/mL in Germany [100]. Another study reported a mean BPA concentration of 8.3 ± 8.9 ng/mL in the amniotic fluid of pregnant women in Japan [101]. In these studies, the maximum level of BPA detected in pregnant women is equivalent to 0.098 µM and 0.083 µM of BPA in the blood plasma and 0.039 µM of BPA in amniotic fluid, respectively. The BPA detected in the serum of occupational workers (0.447 µM BPA), or plasma in pregnant women (0.098 µM and 0.083 µM of BPA) are in the range of 0.05–100 µM of BPA or BPF that we used in our study. Since BPA can easily move across the blood–brain barrier or placenta [94,102,103,104,105], neural stem cells can be exposed to these BPA concentrations. Our data showed that rNSC exposed to lower concentration, 0.05, 0.25 µM, and higher concentration 10, 50, and 100 µM of BPA or BPF not only increased the % differentiation into astrocytes, neurons, and oligodendrocytes, but increased the abnormal or immature neurons. However, morphological changes were observed at higher doses of 50 and 100 µM of both BPA and BPF. Such high doses could occur in accidental ingestion of BPA or BPF in an occupational exposure environment; hence adult neural stem cells could be exposed to 50 µM of BPA or BPF and change the morphologies of astrocytes, oligodendrocytes, and neurons by reducing the cellular arborization.

The differentiation processes of dopaminergic-neurons in the ventral mesencephalon [106,107] and neurons and glial cells in rodents are also regulated by the estrogen–ER pathway [106,108,109]. These observations support our findings, explaining the equivalent neuroendocrine disruptive properties of BPA and BPF. Furthermore, our data suggest that both BPA and BPF enhance the directed-differentiation process of rNSC into astrocytes, oligodendrocytes, and neurons, but result in the failure of cells to acquire the typical morphology characteristics of each cell type. These changes in the three cell types alter the cell ratios, and the morphological differences associated with BPA or BPF exposures may disrupt the neurodevelopment, brain function, and behavioral changes, characteristic of BPA-induced neurobehavioral disorders. Our findings are largely supportive of the earlier findings which suggest that BPF can alter neuronal development, resulting in aberrant brain structure and may predispose individuals to develop cognitive deficits and behavioral changes in young children [102,110]. Several publications showed that functional changes in oligodendrocyte precursor cells and/or oligodendrocytes may also be associated with neuropsychiatric disorders [111,112,113]. BPA exposure during development may also be associated with neurobehavioral changes, such as cognitive deficits, increased anxiety, socio-sexual deficiencies; and neurological disorders such as autism spectrum disorder and attention deficit hyperactivity disorder [114,115,116,117,118,119,120,121,122,123,124]. Some of these effects may be related to BPA acting as a weak estrogen and binding to estrogen receptors within various regions of the brain. Furthermore, Qiu et al. [125], using transcriptional analysis, showed that BPA substitutes BPF and BPS may exercise comparative similar or greater toxicological effects relative to BPA [125,126,127].

The mechanism by which BPA and BPF can affect the differentiation processes, proliferation, changes in morphologies, and arborization are currently not understood, but based on literature, we can hypothesize on the possible mechanisms. Studies have shown that both BPA and bisphenol analogues cross the placenta and the blood–brain barrier [94,102,103,104,105]. These can bind to or act through estrogen receptors (ERα, ERβ, and ERRγ), androgen receptors (AR), thyroid hormone receptors (TH), and signal to its downstream targets (ERK/MAPK, PI3K signalling); binds to β-catenin in Wnt pathway [128,129,130,131] and change Notch signaling [132,133,134]. The role of the Notch signaling pathway in embryonic neural development, in adult neurogenesis, cell fate, stem cell differentiation into neurons, astrocytes, oligodendrocytes, their morphogenesis, and dendritic arborization is well documented [132,133,134]. BPA has been reported to increase cell proliferation and differentiation in neural stem cell cultures and in vivo neural tissues [76,135,136,137]; which supports our findings. Studies have shown that BPA inhibits the activity of γ-secretase in Notch signaling; which results in premature differentiation of NSCs and this led to developmental abnormalities [138]. Decreased activity in secretase also suppresses the down-stream genes ESR1 and PAX6( paired box 6), which are known to play a key role in regulating neuronal differentiation, and neural precursor cell proliferation in mammals [139,140]. Both BPA and BPF decrease neurite length in developing neurons [141]; and activate Notch signaling in rat intestinal epithelial cells and stem cells inducing intestinal development, including cell proliferation and differentiation [133,142]. Further, the Notch receptor (Notch1 and Notch2) expression has been documented in neural stem cells, neurons, astrocytes, and oligodendrocytes [132,143,144,145]. NOTCH1 not only regulates NSC fate and maintenance, but also regulates the dendritic morphology of newborn neurons [132,145]. Inactivation and/or downregulation of Notch signaling results in NSC depletion and induces differentiation into neuronal and glial cells (astrocytes and oligodendrocytes) [108,146,147,148]. Inactivation of highly expressed Hes genes (Hes1, Hes3, and Hes5) in NSC leads to an increase in proneural genes, acceleration of neurogenesis, and premature depletion of NSCs [148,149]. In silico docking studies suggested that BPA downregulates Notch pathway genes Notch-1, Hes-1, and Mib-1 and showed myelin sheath degeneration and neurobehavioral impairment in rats [149]; however, Tiwara et al. (2015) suggested Wnt/B-catenin pathway as one of the targets of BPA [81]. Wnt2 and Ngn2 a downstream target of the Wnt pathway can interact with the Notch signaling pathway in different ways to increase or decrease cell proliferation and neural differentiation processes [148,150,151]. Furthermore, JAG1 signals through NOTCH1 delay in oligodendrocyte differentiation and inhibit oligodendrocyte maturation, thus controlling the timing of myelination during development [132]. BPA enhances dendritic morphogenesis of the cerebral cortex and hippocampal neurons [152,153]. Conditional inactivation of Notch1 in the postnatal hippocampus leads to defects in dendritic morphogenesis [132,154]; conversely, NOTCH1 overexpression promotes dendritic arborization [132,155]. Furthermore, astrocytic Neuroligins control astrocyte morphogenesis [5]. This mechanism could explain the reduction of arborization of astrocyte neurons and oligodendrocytes and their morphological changes caused by BPA and BPF treatment. Considering these facts, the mechanisms through the interaction of Wnt with Notch signaling and estrogen receptor signaling could explain the increased differentiation of rNSC into astrocytes, neurons, and oligodendrocytes, proliferation and their altered morphogenesis caused by BPA and BPF treatment.

## 5. Conclusions

There is paucity of information on the tested chemicals for DNT due to the fact that in vivo guidelines are resource intensive. Further, it is well documented that a wide variety of chemicals have the potential to induce DNT, but 80% of the high production volume chemicals are lacking the required evaluation. Given the worldwide increasing burden neurodevelopmental disorders in children, and animal welfare represented by the 3Rs (reduction, refinement, and replacement of animal methods), there is a paradigm shift to develop in vitro methods to predict neurodevelopmental hazards for regulatory purposes. These methods need to be reliable, relevant, and efficient as screening tools to identify, prioritize, and evaluate chemicals for their potential to induce DNT. With respect to this, we developed in-vitro stem-cell based system to understand the effects of the BPA and BPF on the developmental effects. Using rNSCs, we showed that both BPA and BPF equally affect the proliferation and differentiation of all the three major cell types in CNS. There was a concentration-dependent increase in the number of immature oligodendrocytes, neurons, and astrocytes with both BPA and BPF. There was a concentration-dependent increase in proliferation of neurons, oligodendrocytes, and a minor effect on the lowest concentration on the number of astrocytes, but changes in morphology were observed. Sholl analysis showed that there were changes in cellular processes and dendritic arborization in oligodendrocytes, astrocytes, and neurons. Our in vitro results showed that BPA and BPF equally affect differentiation and proliferation, and hence may affect brain cell development.

## Data Availability

The datasets generated and/or analyzed, including a large number of TIF images and image analysis data during the current study, are available from the corresponding author on reasonable request.
